# Mapping global public perspectives on mRNA vaccines and therapeutics

**DOI:** 10.1038/s41541-024-01019-3

**Published:** 2024-11-14

**Authors:** Jiaxiang Xu, Zhengdong Wu, Lily Wass, Heidi J. Larson, Leesa Lin

**Affiliations:** 1 Laboratory of Data Discovery for Health Limited (D24H), Hong Kong Science Park, Hong Kong SAR, China; 2 WHO Collaborating Centre for Infectious Disease Epidemiology and Control, School of Public Health, LKS Faculty of Medicine, The University of Hong Kong, Hong Kong SAR, China; 3 Department of Infectious Disease Epidemiology, London School of Hygiene & Tropical Medicine, London, UK; 4 Centre for the Evaluation of Vaccination, Vaccine & Infectious Disease Institute, University of Antwerp, Antwerp, Belgium; 5 Department of Health Metrics Sciences, University of Washington, Seattle, WA USA

**Keywords:** Public health, Risk factors

## Abstract

The development and rollout of mRNA vaccines during COVID-19 marked a significant advancement in vaccinology, yet public hesitation to vaccination was prevalent, indicating the potential risk that future mRNA-based medical innovations will fail to be adopted. Utilizing a combined approach of large language models with manual validation and unsupervised machine learning, we conducted a social listening analysis to assess attitudes towards mRNA vaccines and therapeutics on Twitter from June 2022 to May 2023, contrasting online perspectives with data from the Vaccine Adverse Event Reporting System. Our findings reveal widespread negative sentiment and a global lack of confidence in the safety, effectiveness, and trustworthiness of mRNA vaccines and therapeutics, with frequent discussions of severe vaccine side effects, rumors, and misinformation. This underscores the need for targeted communication strategies to foster acceptance of medical treatments and strengthen public trust in order to enhance societal resilience to future health challenges.

## Introduction

Messenger RNA (mRNA) represents a significant advancement in the field of vaccinology, with its crucial role in the expedited development of effective vaccines against COVID-19 highlighting its potential in modern medicine. The two mRNA vaccines, BNT162b2 (Pfizer) and mRNA-1273 (Moderna), have exhibited considerable efficacy in preventing SARS-CoV-2 infection, and significantly lowering the incidence of hospitalization and mortality^1^. Beyond its application in COVID-19 vaccines, mRNA-based therapeutics are being explored for a range of other diseases, including HIV, influenza, and RSV, through preclinical studies and clinical trials^2,3^. This emerging treatment holds great promise for revolutionizing human therapeutic and vaccine development, potentially offering more rapid and flexible responses to emerging health challenges.

Despite extensive research spanning decades into mRNA-based therapies and vaccines^4^ and notable recent advancements, vaccine hesitancy continues to present a substantial challenge. While the expedited development of the mRNA COVID-19 vaccines was largely regarded as a scientific achievement, the unintended consequence of this was a notable drop in vaccine confidence. Compared to traditional vaccines, mRNA vaccines have faced heightened distrust and safety concerns from the public, reflecting wariness towards novel medicines and vaccines, and worries over their emergency use authorization^5,6^. High vaccine hesitancy has been estimated to worsen national outcomes during the pandemic, including elevating mortality rates and prolonging the need for non-pharmaceutical interventions^7,8^.

In the context of vaccine hesitancy, social media has been recognized as a key player in amplifying concerns and misinformation surrounding COVID-19 vaccines during the pandemic^9^. On the one hand, the real-time, high-volume nature of social media platforms like Twitter allows for the immediate capture and analysis of public sentiment, offering a unique lens through which evolving perceptions and concerns can be observed^10,11^. Studies have demonstrated that the monitoring of social media could be critical for public health practitioners and policymakers to better understand and address issues like vaccine hesitancy^12^.

On the other hand, platforms like Twitter, with over 300 million active accounts and 500 million tweets posted daily, have amplified COVID-19 vaccine misinformation^13^, resulting in real-world behavioral consequences like the rejection of vaccines^14,15^. This dual role of social media, as both a valuable research tool and a vector for dangerous misinformation, underscores the need for a nuanced understanding of how public opinions propagate online and their broader implications for societal cohesion.

Existing research has explored public sentiment and attitudes towards COVID-19 vaccines^16–18^, but there is a notable gap in studies specifically focusing on the new mRNA vaccines and therapeutics, including mRNA-based COVID-19 vaccines. This oversight is significant, as the novel nature of the mRNA vaccine platform has contributed to hesitancy surrounding its safety^5^, suggesting that previous studies on traditional vaccine hesitancy do not fully capture the specific concerns associated with mRNA vaccines. Furthermore, traditional sentiment and content analyses based on machine learning require time-consuming sampling and manual data labeling for model training, which hinders rapid processing and analysis. This delay impedes the ability to implement timely responses that could significantly influence policy and communication.

This study employs advanced LLMs to efficiently analyze the vast volume of social media data on Twitter. This optimized approach enables the swift extraction of insights into global sentiment, confidence, and public discourse regarding mRNA vaccines. By facilitating the efficient translation of findings, this method can address key public concerns and inform policy and communication strategies.

## Results

### Validation of GPT-model classification

To validate the accuracy and reliability of the GPT model for classifying tweets, we initially selected a random test set of 6000 tweets from our sample. This test set was coded by two independent coders, with a third impartial coder resolving any discrepancies. We then assessed the performance of the GPT-3.5-turbo model by comparing its classifications with the manually validated labels of these tweets, utilizing both accuracy and F1 score metrics. The results from these metrics confirmed the feasibility of using the GPT model for further analysis (Supplementary Table 1).

### Public confidence in mRNA vaccines & therapeutics

Table 1 conveys the confidence towards mRNA vaccines and therapeutics, which are referred to as ‘mRNA’ in this paper for simplicity. This analysis includes relevant posts on Twitter from June 1, 2022, to May 31, 2023. Overall, of the 740,533 tweets examined, 541,698 (73.1%) discussed aspects of confidence in mRNA. Discussions predominantly revolved around perceived safety (398,218 tweets, 73.4% of confidence-related discussions), followed by trust issues (347,004 tweets, 64.0%), and perceived effectiveness (288,666 tweets, 53.2%). Conversations about perceived importance were less frequent, comprising only 68,749 tweets (12.7%).

**Table 1:** Confidence related to mRNA vaccines and therapeutics on Twitter by Region

Region	Total	African Region (AFR)	European Region (EUR)	Region of the Americas (AMR)	South-East Asian Region (SEAR)	Western Pacific Region (WPR)
Posts, *n*	740,533	16,681	168,942	481,329	13,716	59,865
Relevant to confidence (%)	541,698 (73.1)	12,748 (76.4)	123,634 (73.1)	352,336 (73.2)	9,468 (69)	43,512 (72.6)
Topics among relevant posts^a^, *n*(%)						
Perceived importance	68,749 (12.7)	1505 (11.8)	15,385 (12.4)	44,583 (12.6)	1327 (14)	5949 (13.6)
Important	40,990 (7.6)	811 (6.3)	9045 (7.3)	26,364 (7.4)	996 (10.5)	3774 (8.6)
Unimportant	27,759 (5.1)	694 (5.4)	6340 (5.1)	18,219 (5.2)	331 (3.5)	2175 (4.9)
Perceived effectiveness	288,666 (53.2)	6827 (53.5)	66,524 (53.8)	186,454 (52.9)	5247 (55.4)	23,614 (54.2)
Effective	54,918 (10.1)	820 (6.4)	11,284 (9.1)	35,791 (10.1)	1611 (17)	5412 (12.4)
Ineffective	233,748 (43.1)	6007 (47.1)	55,240 (44.6)	150,663 (42.7)	3636 (38.4)	18,202 (41.8)
Perceived safety	398,218 (73.4)	9530 (74.7)	93,309 (75.4)	257,199 (72.9)	6401 (67.6)	31,779 (73)
Safe	35,368 (6.5)	548 (4.2)	7034 (5.6)	23,611 (6.7)	716 (7.5)	3459 (7.9)
Unsafe	362,850 (66.9)	8982 (70.4)	86,275 (69.7)	233,588 (66.2)	5685 (60)	28,320 (65)
Trust in authority	347,004 (64.0)	8783 (68.8)	78,990 (63.8)	226,960 (64.4)	5162 (54.5)	27,109 (62.3)
Trust	37,087 (6.8)	854 (6.6)	7619 (6.1)	24,402 (6.9)	847 (8.9)	3365 (7.7)
Distrust	309,917 (57.2)	7929 (62.1)	71,371 (57.7)	202,558 (57.4)	4315 (45.5)	23,744 (54.5)

^a^Topic percentages are calculated relative to posts relevant to confidence per region.

There is a predominantly negative sentiment in discussions about perceived safety, trust in authority, and perceived effectiveness. Specifically, most tweets related to safety perceptions regarded mRNA as unsafe, totaling 362,850 tweets (66.9%). Discussions about trust in authority were mainly marked by distrust, accounting for 309,917 tweets (57.2%). In the category of perceived effectiveness, 233,748 tweets (43.1%) perceived mRNA as ineffective. In contrast, the topic of perceived importance saw a larger proportion of tweets (40,990, or 7.6%) recognizing its importance, outnumbering those who perceived mRNA as unimportant (27,759 tweets, 5.1%).

Despite an overall low confidence in mRNA as reflected in social media discussions, an analysis of tweets across five regions reveals that confidence within the South-East Asian Region (SEAR) and the Western Pacific Region (WPR) is comparatively more positive. In the SEAR, the proportion of tweets characterizing mRNA as “Important,” “Effective,” “Safe,” and “Trust” constitute 10.5%, 17%, 7.5%, and 8.9% respectively. Correspondingly, these measures are 8.6%, 12.4%, 7.9%, and 7.7% in the WPR. Although this does not depict an entirely optimistic scenario, these numbers are nevertheless higher than those observed in the other three regions, indicating a regional variation in the public’s confidence in mRNA.

### Sentiment analysis overall

**Fig. 1:**
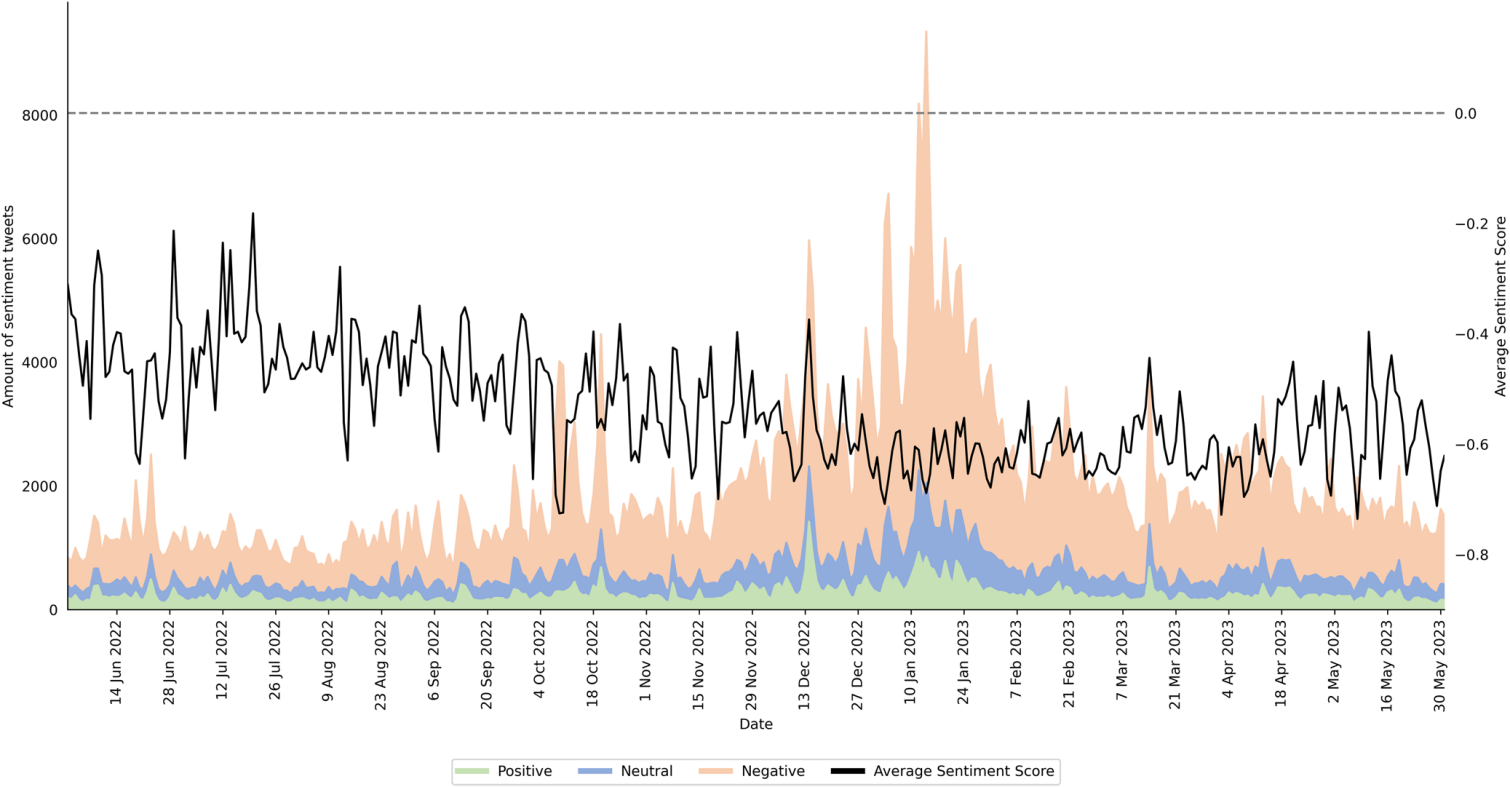
Daily sentiments towards mRNA over time (*n* = 740,533). Daily frequency of positive, neutral, and negative sentiments towards mRNA and the average sentiment score on Twitter during the study period, June 1, 2022 to May 31, 2023.

The overall sentiment trend towards mRNA vaccines and therapeutics was measured in tweets shared between June 1, 2022, to May 31, 2023, spanning 364 days (Fig. 1). The data reveal that negative sentiment tweets predominated, comprising 69.5% of the total. This proportion is notably higher compared to positive (13.01%) and neutral sentiments (17.49%). The average daily tweet count was 2,028.85, peaking at 9,346 tweets on January 14, 2023, and reaching a low of 672 tweets on September 11, 2022. The daily sentiment score for mRNA-related tweets ranged from -0.736 (May 8, 2023) to -0.181 (July 20, 2022). These figures suggest an overall negative sentiment during the period studied.

### Geographical sentiment analysis

**Fig. 2:**
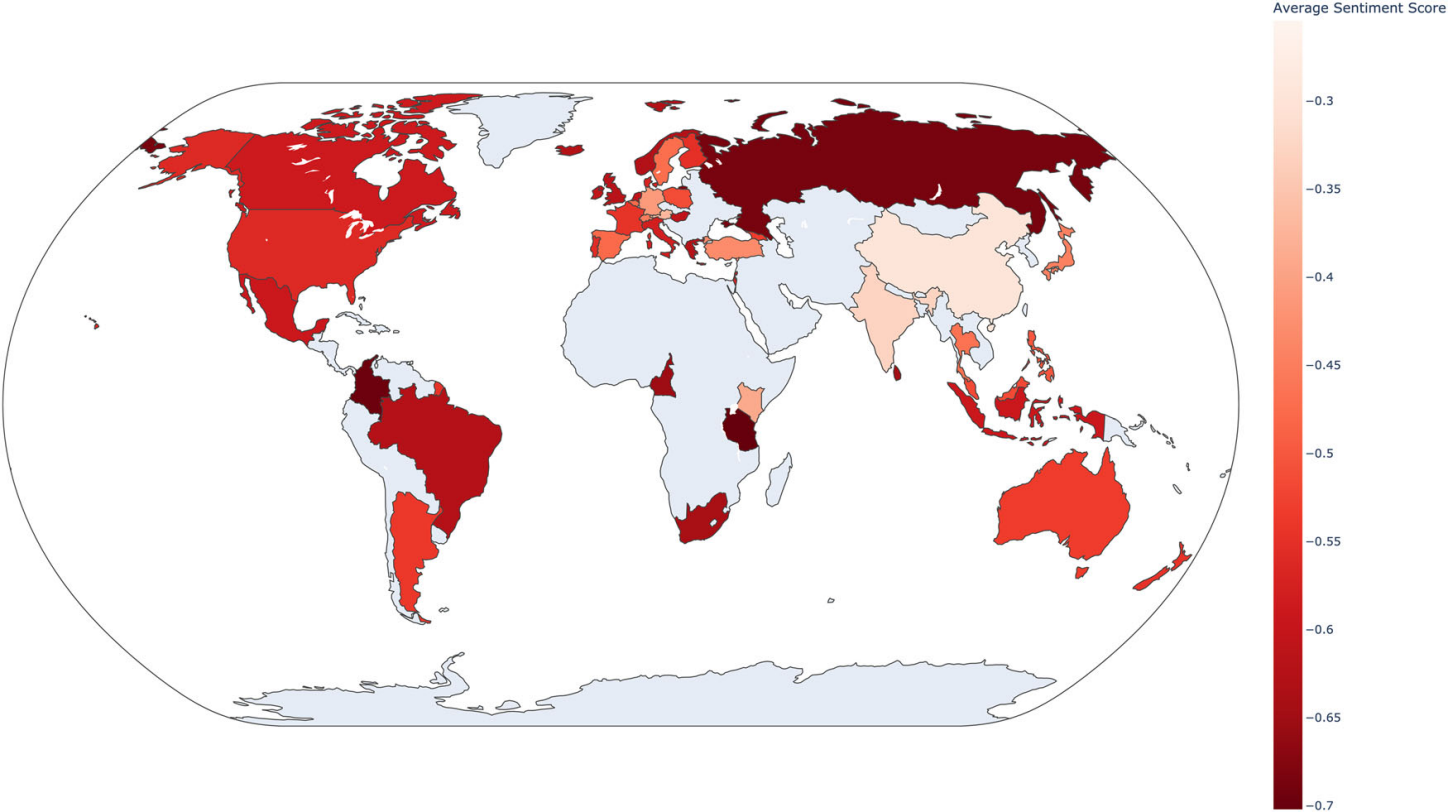
Average sentiments towards mRNA by country. Average score of sentiments towards mRNA across 44 countries during the study period. Gray shaded regions represent countries not present in the sample.

The sentiment analysis across 44 countries in various regions indicates that negative emotions are prevalent across all surveyed territories. As delineated by the heatmap in Fig. 2, the mean sentiment scores for all nations remain below 0. In congruence with the distribution of confidence, the SEAR and the WPR register relatively higher mean sentiment scores of −0.39 and –0.51, respectively. These scores modestly exceed those of the Region of the AMR at –0.56 and the EUR at −0.58, with the AFR recording the lowest mean sentiment score of −0.64.

### Sentiment analysis by country

**Fig. 3:**
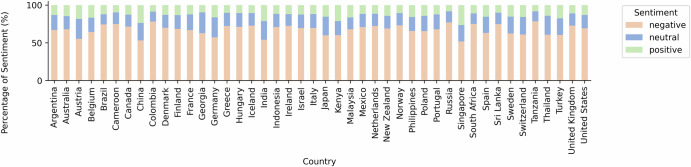
Breakdown of sentiments towards mRNA by country. Percentage of positive, neutral, and negative sentiments towards mRNA across the 44 included countries in the study period.

Figure 3 illustrates the breakdown of tweet sentiments across the 44 different nations contained in our sample, revealing a diverse range of attitudes toward mRNA. Negative sentiments were observed across all countries involved in the study. Notably, Tanzania, Colombia, and Russia exhibited the most negative attitudes, with negative sentiments comprising 78.45%, 78.18%, and 76.95% of the tweets, respectively. Conversely, countries like Singapore, India, and China present relatively less negative sentiment towards mRNA. In Singapore, 51.86% of tweets were negative towards mRNA, while India and China followed closely with 53.17% and 54.07%, respectively. Despite this, the overarching sentiment in these countries remained predominantly negative. Furthermore, the statistical analysis of average sentiment scores revealed significant variances across different countries (ANOVA, *F* = 93.875, *p* < 0.001), suggesting that the observed differences in public sentiment are not random but instead reflect distinct national perspectives.

**Fig. 4:**
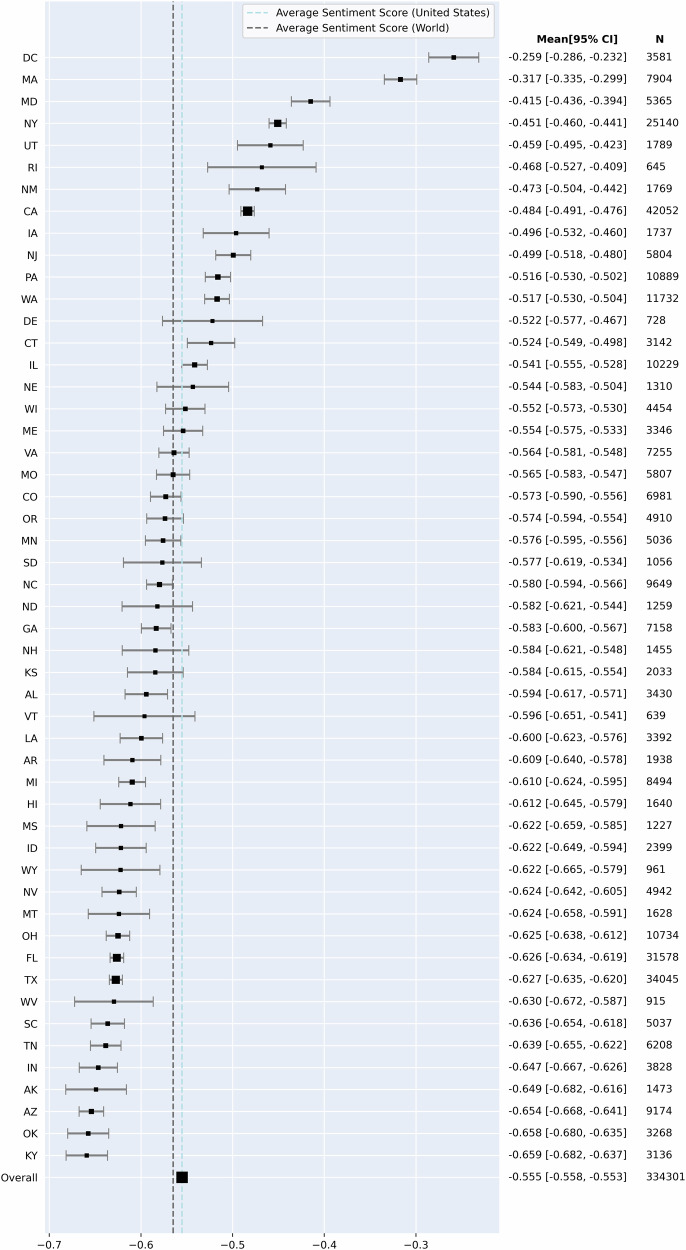
Average sentiments towards mRNA in the United States (*n* = 389,178). Average sentiment score and 95% CI by US state, including Washington D.C, compared to the national and global average.

Given that tweets from the United States comprise the highest proportion (389,178/740,533, 53%), we conducted an analysis of the sentiment across various states in the U.S. A total of 334,301 out of 389,178 tweets, from users with detailed state-level geographic locations, were analyzed. Figure 4 visualizes the mean sentiment scores for each state, along with their respective 95% confidence intervals (CIs). The overall mean sentiment score for US-based tweets was −0.555 [−0.558, −0.553], which, while slightly higher than the global average, indicates a generally negative sentiment nationwide. Washington DC showed the least negative sentiment, averaging −0.259 [−0.286, −0.232], while Kentucky had the most negative sentiment, at −0.659 [−0.682, −0.637]. A significant disparity in sentiment across states was evident (ANOVA, *F* = 88.657, *p* < 0.001). Additionally, the analysis explored sentiment variations between states based on political affiliation, with the Point-Biserial Correlation Coefficient revealing a significant correlation between sentiment scores and political lines (*r* = −0.468, *p* < 0.001).

### Adverse event analysis on Twitter v.s. VAERS

**Fig. 5:**
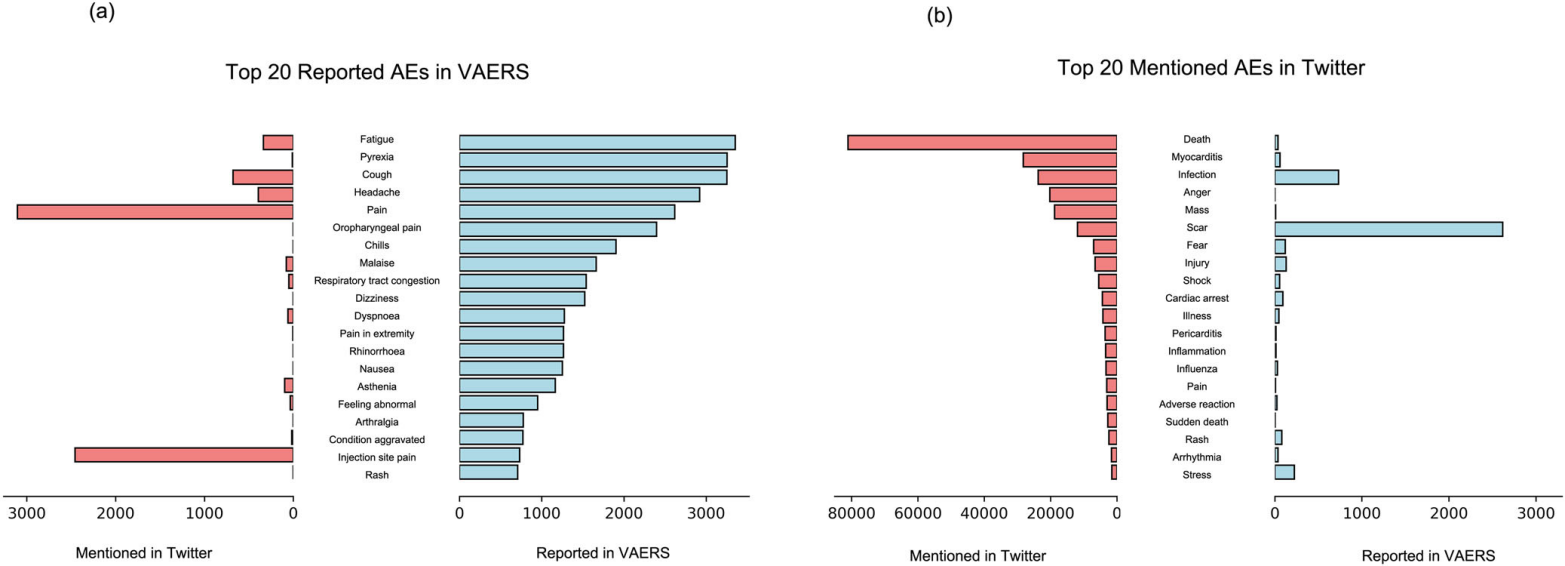
Top 20 most frequently mentioned adverse events on VAERS and Twitter. Frequency of the 20 most mentioned adverse events shared in relation to the mRNA COVID-19 vaccine in VAERS (**a**) compared with the Twitter (**b**) sample.

Figure 5a presents the top 20 post-vaccination symptoms reported in VAERS based on the frequency. The most frequently noted symptoms included ‘fatigue,’ ‘pyrexia,’ ‘cough,’ ‘headache,’ and ‘pain,’ indicating that from a clinical perspective, the most common vaccine side effects are typically mild. These terms primarily highlight physiological responses to vaccination and are characteristic of the specific and technical language used in medical reporting. Apart from ‘pain’ and ‘injection site pain’, the occurrence frequency of other symptoms on Twitter is comparatively lower.

In contrast, the top 20 symptoms mentioned by Twitter users within the symptoms list extracted from VAERS (Fig. 5b) painted a notably different landscape of public sentiment and concern regarding mRNA vaccine side effects, one that does not accurately reflect documented adverse reactions to the vaccine. The discourse was dominated by terms associated with severe and emotionally charged reactions, with ‘death,’ ‘myocarditis,’ and ‘infection’ being the most prominent. The frequency of terms like ‘anger,’ ‘fear,’ ‘shock,’ and ‘stress’ indicated a heightened emotional response from the public, signifying apprehension and distress surrounding mRNA vaccines. Interestingly, terms such as ‘cardiac arrest,’ ‘pericarditis,’ and ‘arrhythmia’ were observed, which are rare side effects and were prominently featured in public discourse.

### LDA topic modeling

**Fig. 6:**
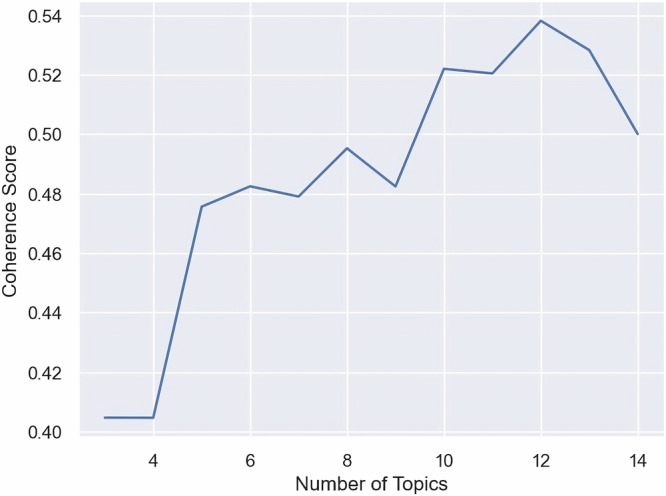
LDA topic model coherence by topic number. In the range of 3 to 15 topics, *n* = 12 topics achieved the highest coherence score of 5.3816 and was selected as the final LDA model for topic modeling of mRNA discussions.

Determining the optimal number of topics (*k*) is crucial in Latent Dirichlet Allocation (LDA) topic modeling, significantly impacting the model’s accuracy and the clarity of the derived topics^19^. Insufficient topic numbers can lead to the omission of critical themes, whereas an excess may obscure focal points^20^. In our study, we defined a candidate range of 3 to 15 topics and performed coherence score evaluations to determine the clarity and pertinence of topics within this spectrum. Based on the highest coherence score achieved (Fig. 6), we selected 12 topics. For each identified topic, the researchers selected 5 sample posts with the highest LDA output percentage and five random posts with corresponding dominant topics for manual review. The topic interpretability was manually assessed by reviewing the selected data points. A topic was designated a specific name (Main Theme) when the sample data points, all sharing the same dominant topic, exhibited content uniformity.

The 12 topics generated from LDA topic modeling effectively cast light upon the expansive spectrum of public discourse pertaining to mRNA vaccines and therapeutics, uncovering a multifaceted tableau of sentiment and thematic elements (Table 2). These themes range from specific health concerns to broader distrust in mRNA, with the general sentiment resonating with findings from prior analyses: a predominant share of the discourse was imbued with negative sentiments.

**Table 2:** Percentages of tweets related to each mRNA topic

Main theme	Top terms contributing to the topic model	Total tweets (%)	Example tweet
Hesitancy and Conspiracies	jab, injection, shot, lie, government	16.8%	• “Still Lying about the mRNA vaxx!! Needs a Warning label attached! There’s no way in hell I’d take another vaccine, much less a mRNA experimental one. And I’m not older, nor do I have a preexisting condition.” • “New Zealand Government’s own stats show the highest percentage of #COVID deaths were among the vaccinated and boosted. These jabs are a giant fraud, not safe, nor effective. #VaccineDeath #vaccine #COVIDVACCINE #PfizerLiedPeopleDied #mRNA #CrimesAgainstHumanity #Nuremberg2” • “Argentina—Mother of a 3 year old that died after the cIot shot says the government constantly pressuring has made her vax her child. She says she is twice jabbed. Her child only had one. Bringing to light the hidden realities, waking up the sleepers.”
“Experimental Gene Therapy” Claims	gene, therapy, COVID, experimental, long	16.2%	• “@WHO Please produce a placebo safety test for the vaccines you push. Why are vaccine manufacturers absolved from product liability. Every vaccine product insert label lists death as a side effect. The COVID shot is mRNA therapy not a vaccine” • “You are mixing up US and EU definition of gene therapy. US = editing DNA using CRISPR for therapy EU = using nucleic acids for therapy. The vaccine is not considered gene therapy in the US because it doesn’t edit DNA. It is considered gene therapy in the EU because it has mRNA” • “You jabbed children with an experimental mRNA they “did not” need and compromised their immune systems. Now this respiratory issue is the result ‘on mass’.”
Biological Mechanism	protein, spike, cell, virus, DNA	11.2%	• “mRNA vaccines = spike proteins = blood clots = death” • “#CovidVaccines (mRNA based) are not vaccines. They are a form of gene therapy where modified mRNA (encased in toxic lipid nanoparticles that travel all over the body) carries the genetic code to make cells produce synthetic spike protein. There is no off switch.” • “Previous vaccines used dead or weakened viruses to stimulate the immune system. C19 V uses synthetic mRNA spike proteins which is not easily degraded n can induce inflammations. The lipid nanoparticles r able to enter the blood-brain barrier to cause brain fog and prion diseases.”
Adverse Events	death, covid, risk, myocarditis, cause	10.9%	• ”Anyone who took it has a higher risk of death. Two young boys in Connecticut died days after mRNA vax with autopsies showing acute myocarditis.“ • “This Danish study confirms a substantial risk of myocarditis/pericarditis in young males following mRNA vaccination, even in comparison to the risk of similar complications following COVID infections. So are mRNA vaccines really “safe and effective”, as it was claimed?” • Covid mRNA vaccines cause myocarditis and pericarditis, but Covid infections do not. Huge 500k+ participant study. We were lied to, again. How many times have experts told us that post-Covid myocarditis is worse? This is so disheartening--pun intended.
Safety Concerns in Children	booster, covid, dose, shot, children	8.6%	• “The American Heart Association published a new study which found that 98% of all cases of Myocarditis among children are due to the mRNA Covid-19 injections.” • “Pfizer clinical trials of the mRNA shots showed vaccinated children get elevated rates of a certain respiratory virus, RSV. Now sick children with this virus are filling hospital ERs. In the meantime, CA is forcing the mRNA shots on school children. #StoptheShots” • “It doesn’t matter who has vaccinations before Covid, this was 1. An mRNA delivery that is new. 2. Vaccines for children are not enforced as ‘take it or you can’t go to school.’ 3. Vaccines are not suitable for ALL people.”
Global mRNA Vaccine Efforts	development, China, new, research, world	8.5%	• “As it happens, countries that were only able to get sufficient doses of the MRNA vaxxes initially- such as Indonesia- have boosted w MRNA vaxxes. China and India are developing their own MRNA vaccines and has no objection to the method.” • “BioNTech Expands Global Footprint by Acquiring GMP Manufacturing Site to Establish First mRNA Facility in Singapore” • “China has shown that it can develop non-mRNA vaccines without too much risk of side effects for the population. Example SINOVAC and SINOPHARM… Health policy must protect the people, not make them sick as the West does”
mRNA Tech in Food Supply	food, supply, animal, livestock, meat	7.1%	• “Any meat (or any byproduct) should be clearly labeled as a mRNA product, so consumers can be aware of this. Should be Federal Law EVERYWHERE! I certainly do not plan on buying any product with MRNA in it.” • “Australia intends to vax all cattle by the end of the year with MRNA. It will be in milk & cheese. Bill Gates wants to get it into plants as well & Jacinta Ardern threatened to put it in the water supply if enough people did not take the shot. Awake yet?” • “Bill Gates is putting mRNA is his livestock. The same spike protein killing people will be injected in the food you eat, so you join the club.”
Controversies in Scientific Community	Twitter, doctor, inventor, scientist	6.6%	• “I don’t see how someone who claims to have “invented mRNA technology” could be so stupid. The amount of misinformation circulating on the internet from “mRNA” trending on Twitter is a direct result of Elon Musk’s influence on the platform. This is a public health risk.” • “@elonmusk pls make sure all of Dr. Malone’s interviews are not blocked on this platform. I have about a dozen I’d like to post but will start with this quote from the inventor of mRNA technology. Sidenote: Anyone ever gotten a mild case of typhoid fever? Polio? Did’t think so.”
Distrust in Big Pharma	Pfizer, safe, shot, moderna, medical	4.1%	• “How many of those that took Pfizer and Moderna were aware that they actually used a new with no long-term safety data that injects synthetic mRNA code that instructs the body to produce synthetic spike protein?” • “Pfizer’s Latest Flu Vaccine Is mRNA Based, Avoid At All Costs.” • “They’re going to be using MRNA for everything….; A Terrible Idea—Pfizer, Moderna Developing Combined mRNA Omicron-Flu Vaccine”
Evidence-based Vaccine Concerns	harm, evidence, datum, safety, death	3.8%	• “Lead author of peer-reviewed research re-analyzing Pfizer & Moderna trials on mRNA vaccine @JosephFraiman calls for immediate suspension of jab due to serious harms. ‘We have conclusive evidence that the vaccines are inducing sudden cardiac death’.“ • “I worked with mRNA and understood exactly how the COVID shots work. Following the concerning safety signals/lack of efficacy data did not prevent me from being fired. I’m an ‘extremist’ according to the PM.” • ““Changing evidence?” Loads of us non-medical experts knew it was a disaster waiting to happen back in Dec 2020. If you didn’t know the risk from an mRNA gene therapy, why not? If you did know the risks, why advise people to take it? None of this makes sense.”
Blood-related Concerns	heart, immune, blood, clot, attack	3.5%	• “TRAGIC: Claire Bridges was a 21-year-old model when she received the mRNA vaccine. Clair ended up having legs amputated due to blood clots and now suffers from myocarditis & kidney failure. #diedsuddenly” • “mRNA injections destroy your natural immune system, causing all sorts of diseases along with heart problems and blood clots. Lancet study and other studies prove the mRNA injections are dangerous and deadly.” • “Study from the University of Basel (Switzerland) detects heart muscle cell damage in at least 3 out of 100 persons inoculated with a mRNA booster vaccine shot.”
mRNA and Cancer	cancer, news, medium, death, factor	2.7%	• “Cancer is exploding Worldwide. It is believed among other things, the spike proteins from mRNA injections shut-down a cellular process called APOPTOSIS, which VERY simply put, tells a damaged cell to Die before it becomes Cancerous, so Immune System cells can carry them away. ” • “Cells Don’t Lie: Cancer is on the Rise Due To mRNA Injections.” • “Y’all are dumb af. mRNA is something that’s literally in all of us. mRNA in the COVID vaccines does not cause cancer.”

The predominant theme identified was “Hesitancy and Conspiracies,” accounting for 16.8% of the discussion. This theme was characterized by terms such as ‘jab’, ‘lie’, ‘government’, and ‘experimental’, reflecting widespread skepticism and misinformation. Tweets under this theme primarily focused on distrust towards the government, with discussions suggesting that those involved with mRNA were “lying” about their implications and safety. The theme “Experimental Gene Therapy’ Claims”, encompassing 16.2% of the tweets, captures significant public unease. This concern largely stems from the rapid development and emergency authorization of COVID-19 mRNA vaccines. The use of the term “experimental” in discussions frequently signifies a deep-seated mistrust and skepticism among individuals regarding the safety and efficacy of these vaccines.

Furthermore, “Biological Mechanism” and “Adverse Events” themes, representing 11.2% and 10.9% of the discussions, respectively, incorporate extensive use of scientific terms such as ‘protein, ‘ ‘spike, ‘ ‘cell, ‘ ‘DNA, ‘ and ‘myocarditis.’ While these discussions employ technical language that might appear authoritative, they often propagate misinformation, leading people to accept false notions as facts. This prevalent misuse of scientific language underlines the critical need for accurate and accessible scientific communication. It is essential to clearly explain how mRNA-based therapies and vaccines function and to effectively address public concerns, ensuring that public discourse is informed by correct and verified information. Following this, the theme “Global mRNA Vaccine Efforts,” which accounts for 8.5% of the discourse, highlights a global acknowledgment of the efforts to combat the pandemic through scientific advancements in vaccine development. This theme contrasts with those that feature misinformation, showcasing a more positive perception of international collaboration and scientific progress.

**Fig. 7:**
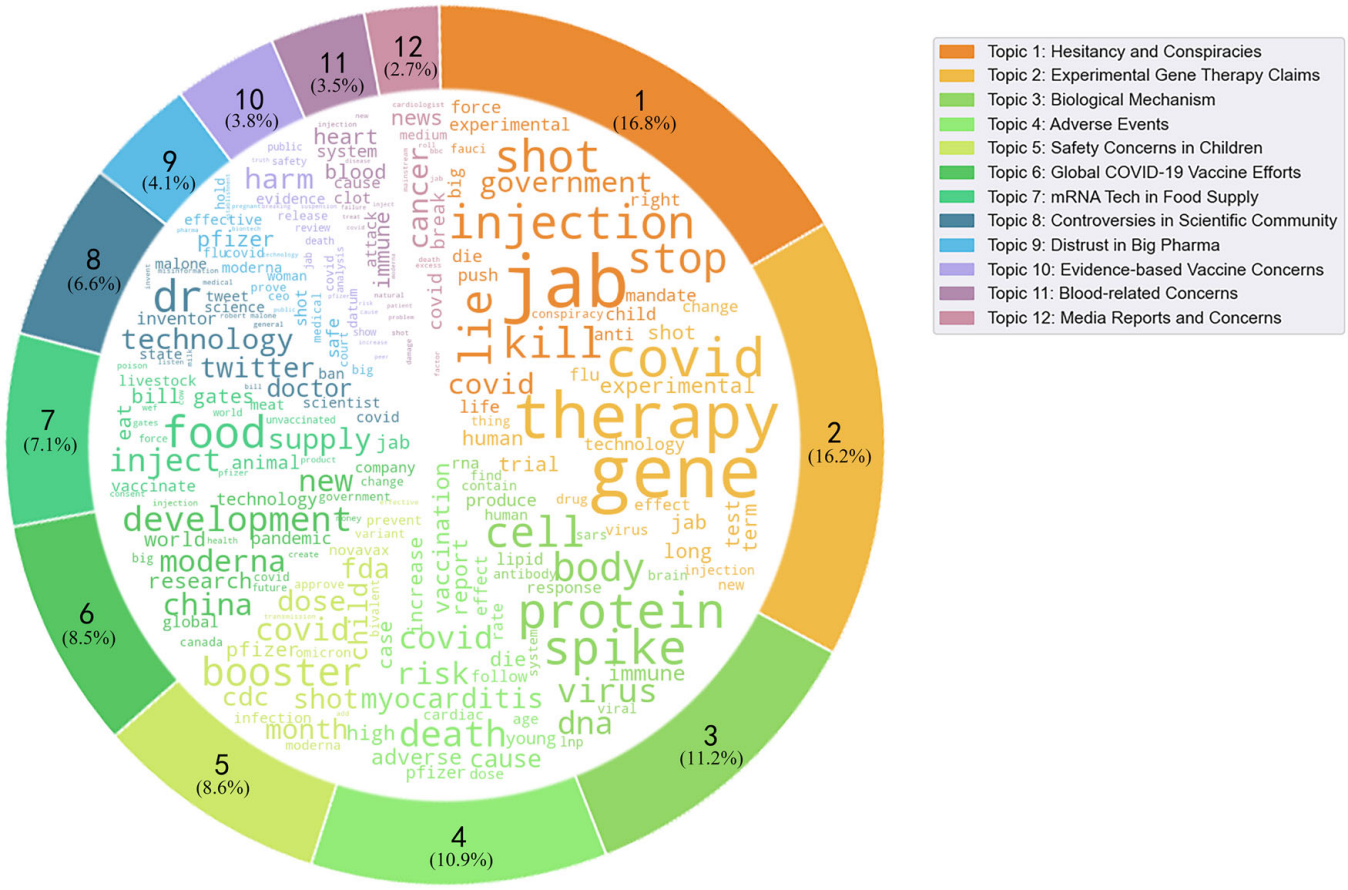
mRNA-related discussion topics and keywords on Twitter. 12 dominant discussion topics surrounding mRNA and the most frequent words observed in each topic based on the LDA model.

Additionally, the remaining themes delve into more specific aspects of mRNA discussed by users, such as “Controversies in Scientific Community,” “Distrust in Big Pharma,” “Blood-related Concerns,” and “mRNA and Cancer.” Notably, the theme “mRNA Tech in Food Supply” comprises 7.1% of the discourse, where a significant number of discussions revolve around the application of mRNA vaccines in livestock. There is prevalent concern among users that consuming meat from these animals might alter human genetics. This particular topic underscores the extensive range of misinformation surrounding the implications of mRNA, extending beyond just COVID-19 vaccines. Figure 7 presents word clouds for the 12 dominant topics, complementing Table 2 by visually demonstrating more relevant terms for each topic and enhancing our understanding of their thematic content.

## Discussion

In this study, we leveraged LLMs to assess the sentiment and confidence towards mRNA vaccines and therapeutics shared on Twitter. Additionally, we utilized LDA Topic Modeling to detect various aspects of the discussions surrounding mRNA. Our findings indicate that confidence in mRNA is generally low, with negative sentiments being more prevalent than those reported in previous social media-based studies of attitudes toward COVID-19 vaccines in general. By comparing discourse on Twitter with VAERS data, we observed that Twitter users widely discuss severe side effects that are reported infrequently in VAERS official records.

Although prior studies from 2020 to 2021 examining attitudes towards general COVID-19 vaccines acknowledged the existence of concerns and negative sentiments on social media, they generally reported a higher proportion of positive sentiments compared to negative ones^20–22^. This positive sentiment can be attributed to the excitement and hope surrounding the introduction of vaccines during the early pandemic phase, when effective therapeutics were not yet available, and the public was eager to return to pre-pandemic normalcy. A 2021 Twitter sentiment analysis indicated that a majority of tweets—54.53% across American metropolitan areas—exhibited positive sentiments regarding the COVID-19 vaccine^23^. One analysis of tweets about the COVID-19 vaccine published in Australia in 2021 found that 66% exhibited negative sentiments, a finding similar to our results but still lower than the 69.5% negativity we observed^24^. In general, our research indicates that public sentiment toward mRNA vaccines from 2022 to 2023 was lower than overall sentiment toward COVID-19 vaccines in prior years, reflecting the challenges associated with the novelty and ongoing misinformation about mRNA technology. After vaccination, concerns regarding various side effects and the subsequent spread of misinformation began to influence public perception, contributing to a decline in confidence over time.

As previous research has shown, the COVID-19 mRNA vaccine initially saw higher public sentiment during the early stages of its rollout, which, in our current research, is found to be lower compared to earlier periods. A potential reason is reflected in our topic modeling result, which revealed a significant abundance of skepticism and misinformation about mRNA vaccines, contributing to the public’s negative sentiment. Our analysis identified that the two most prominent topics, constituting 16.8% and 16.2% of the discourse, unveil a deep-rooted governmental distrust and apprehension regarding mRNA vaccines. In our sample, these vaccines were often labeled as ‘experimental’ due to concerns about their safety and unproven long-term effects. Additional discussions include skepticism about the biological mechanisms of the vaccines, anxiety over potential adverse effects, and contentious debates within the scientific community, further supporting the ‘novelty penalty’ against mRNA vaccines. It is established that the spread of misinformation or rumors on social media is significantly associated with vaccine hesitancy^25^. This frames the challenge in boosting confidence in mRNA technology, which has applications far beyond COVID-19, including vaccines for other infectious diseases and therapeutic vaccines for cancer^26,27^. While progress in mRNA development shows promise, widespread negative opinions and misinformation online highlight the need to create narratives that build confidence and acceptance, allowing mRNA to reach its full potential in healthcare and disease prevention.

Despite a marginal win in support for the importance of mRNA vaccines over unimportance, persistent concerns regarding safety, effectiveness, and trust continue to prevail. Through comparisons across included regions and countries, our study also suggests that hesitancy is not merely a reflection of individual concerns but is likely influenced by broader social, cultural, and political factors that result in distinct regional differences. Notably, higher levels of positivity and confidence were observed in the SEAR and the WPR. Significant variations in sentiment across various states in the U.S. further underscore the need for more detailed analysis and region-specific approaches to understand and address regional hesitancy toward mRNA vaccines and therapeutics. Such geographic variation in mRNA confidence highlights the necessity for nuanced, context-sensitive public health interventions that resonate with the specific values, beliefs, and socio-political landscapes of different populations. Research has shown that the pervasive lack of understanding about mRNA also fosters negative sentiments^28^. Data from the Global Listening Project indicates that less than half of the US public has heard a great deal or a fair amount about mRNA vaccines (unpublished data from the Global Listening Project presented at the Global mRNA Conference Boston 2023 by Heidi J Larson). To address this, a more proactive public health presence on social media could be critical. Health systems should closely monitor public discourse and concerns expressed on platforms like Twitter and provide more scientifically robust and evidence-based information to the public. This approach will not only help inform policies related to public health responses but also enhance overall public understanding and acceptance of mRNA vaccines.

Although VAERS is a U.S.-based system and the Twitter data analyzed in our study were derived from users in 44 different countries, the inclusion of VAERS data was intended to serve as a benchmark for understanding how vaccine-related discussions unfold on a global platform such as Twitter. Therefore, our comparisons aim to reveal different patterns in which adverse effects are reported in the real-world and discussed across various global regions on Twitter, rather than providing a direct statistical correlation. Our analysis revealed a stark contrast between the discourse on Twitter and reported official data from VAERS, with Twitter disproportionately discussing extreme side effects that are reported very infrequently in health system records. The VAERS data presents symptoms such as fatigue, pyrexia, cough, headache, and pain, which are common and generally mild post-vaccination reactions that typically resolve on their own^29^. However, the Twitter data exposed concentrated discussions on more severe adverse effects like death, myocarditis, and infection, which, despite being officially recognized as side effects, have a low incidence rate^30^. This discrepancy underscores the influential role of social media in polarizing health-related issues and magnifying concerns, often distorting the public’s perception of reality. Understanding and addressing this divergence is crucial for health authorities to ensure accurate public awareness and mitigate unwarranted fears stemming from misrepresented information on social media.

Upon rigorous validation, we have also ascertained the feasibility of employing LLMs for conducting refined sentiment and content analyses within the context of our confidence framework. This validation not only fortifies the credibility of our analytical approach but also marks a significant step forward in social listening research methods within public health domains. The deployment of LLMs in our study underscores the transformative potential of these advanced computational models to analyze public sentiment and concerns quickly and effectively, without the need for human-labeled data. This capability is particularly crucial during public health emergencies, where LLMs can provide rapid insights, enabling policymakers to respond more swiftly to evolving situations, and thereby facilitating more effective health communication strategies and intervention.

Our study also has several limitations. First, analyzing social media data, particularly from Twitter, means that the platform’s users tend to be younger and more tech-savvy, potentially excluding older adults or those without digital access. Consequently, the sample may not fully represent broader public attitudes. Engagement dynamics on social media can also introduce bias, as emotionally charged or controversial content may generate more attention. In this study, we used a dataset consisting only of original tweets and retweets containing original comments. This approach helps minimize the impact of automated accounts (bots) that may disseminate either pro- or anti-vaccination content. Nonetheless, the potential influence of bots remains a limitation, as studies have shown that bots can play a notable role in creating and spreading health-related misinformation, potentially skewing perceptions of public sentiment^31–33^. Caution is needed when generalizing these results to the entire population.

Additionally, the GPT-3.5 turbo model used for sentiment and confidence analysis may introduce biases due to its opaque nature. While LLMs are increasingly adopted in research, their reliability requires further validation. In this study, we manually coded data to assess the feasibility and accuracy of the GPT-3.5 model within our framework, but future research should extend this validation across various datasets and analytical contexts. Moreover, as an early warning system, while very important in monitoring vaccine safety, VAERS reports alone cannot be used to determine if a vaccine caused or contributed to an adverse event or illness. Thus, our comparison does not establish the actual frequency or severity of specific symptoms. Our findings just highlight discrepancies between side effects discussed by Twitter users and those reported in official reporting sources. Further clinical investigations and rigorous studies are needed to determine causality and accurately quantify the incidence and severity of vaccine-related adverse effects.

## Methods

### Twitter data collection

By mid-2022, a significant portion of the global population had been vaccinated with at least one dose of a COVID-19 vaccine, according to data provided by Our World in Data^34^. By selecting “People vaccinated” and “Cumulative” on the referenced website, we found that the vaccination rate was 65.06% as of June 1, 2022, marking a pivotal juncture in the pandemic’s trajectory. This period from mid-2022 to May 2023 also witnessed a paradigm shift in COVID-19 management strategies, characterized by a gradual easing of lockdowns and social distancing measures in various countries. Notably, the World Health Organization (WHO) formally lifted the COVID-19 Public Health Emergency of International Concern status on May 5, 2023^35^, further underscoring the transition into a new phase of post-pandemic management. These developments provide a dynamic field to detect public perception and sentiment regarding mRNA vaccines and therapeutics.

To capture these changes, we selected the one-year period from June 1, 2022, to May 31, 2023, for our study. During this timeframe, the public discourse around mRNA vaccines was likely to reflect both the broad discussions of vaccinations and the impact of changing public health policies. Moreover, this time period offers a comprehensive understanding of the global discourse around mRNA, capturing more stable public sentiment and opinions as the world has had over a year to familiarize itself with this approach to making vaccines. Using “mRNA” as the keyword, we collected tweets via the Meltwater platform^36^, an extensive digital monitoring tool. We chose the search term “mRNA” and its variants, such as “mrna”, “Messenger RNA” to specifically capture discussions related to the mRNA vaccine or mRNA itself. Although “mRNA” is a scientific term, public awareness and discussion about mRNA increased significantly during the COVID-19 pandemic. People were more inclined to use the term “mRNA” or hashtags like #mRNA, #mrna, to engage in discussions specifically about mRNA, its origins, principles, the COVID-19 vaccines developed using it, and its potential applications in various fields. The dataset comprising 4,094,987 tweets, included details such as account name, content, posting time, follower count, and engagement metrics.

### Twitter data cleaning

To obtain the appropriate content for analysis, duplicates, retweets, quotes without original comments, and unrelated posts (e.g., spam tweets, advertisements) were removed^37^. Tweets were restricted to users with specified locations for subsequent geographical and country-based descriptive analysis. Additionally, the analysis was confined to tweets in English to ensure consistency in language processing and interpretation. Following data cleaning and excluding countries with less than 1000 tweets, our analysis encompassed 740,533 tweets across 44 countries.

### VAERS data collection

The Vaccine Adverse Event Reporting System (VAERS), managed by the Centers for Disease Control and Prevention (CDC) and the Food and Drug Administration (FDA), is a vaccine safety monitoring system that accepts and analyzes reports of post-vaccination side effects. In this study, we collected VAERS data associated with the BNT162b2 and mRNA-1273 vaccines reported in the same period as our Twitter data (*N* = 134,596). The Adverse Event Reporting dataset encompasses 35 attributes, such as ID, date, age, gender, and a brief description of the side effects, including up to five symptoms. A total of 4,331 unique symptoms were reported during the period. We then created a list ranking each symptom reported by its frequency of occurrence and subsequently compared this data with our Twitter findings. It should be noted that VAERS data, derived from submissions by healthcare professionals and individuals, does not intrinsically establish a causal relationship between vaccination and the reported symptoms^38^.

### Confidence and sentiment analysis framework

To explore emerging public sentiment towards mRNA vaccines and therapeutics, we used the GPT-3.5-Turbo model to rapidly classify our Twitter sample on several dimensions of confidence. In the field of natural language processing (NLP), GPT-3.5 is one of several emerging LLMs. These sophisticated models have shown remarkable text processing and generation ability powered through extensive training on diverse text corpora^39,40^. This includes applications in sentiment classification and content analysis of text. Furthermore, the implementation of zero-shot or few-shot learning methodologies, often facilitated through prompt-based approaches, enables LLMs to process information swiftly. This capability significantly reduces the time and resources required for manual data annotation and model training, thereby enhancing efficiency in deploying solutions. We sought to use GPT-3.5 to label all tweets in our sample across four measures of mRNA confidence and one overall sentiment label.

The WHO Strategic Advisory Group of Experts on Immunization (SAGE) identified three overarching divers of vaccine hesitancy-Confidence, Convenience, and Complacency^41^—among which we selected ‘Confidence’ to frame our classifications of Twitter attitudes towards mRNA. Within the broader dimension of confidence, we identified four key categories of analysis: perceived importance of mRNA vaccines and therapeutics^42,43^, perceived effectiveness, perceived safety, and trust in authority. This model has been validated in prior research on COVID-19 vaccines, proving effective in analyzing sentiment and confidence trends on social media^44–46^. Within each classification, tweets were categorized into one or multiple confidence categories or remained uncategorized if irrelevant to all categories. In addition, the overall sentiment variable classified attitudes towards mRNA as positive, neutral, or negative. These variable definitions were refined in a prompt that was passed to the GPT-3.5 turbo model to label all tweets in our sample (Supplementary Note 1). Upon classification of all tweets in the sample, we coded positive, neutral, and negative post sentiments to 1, 0, and −1, respectively, to facilitate the computation of average sentiment scores across various groups in our sample.

### Adverse event analysis on Twitter v.s. VAERS

Employing keyword extraction, a crucial technique in text mining, we compared the VAERS dataset with Twitter data. We first retrieved the symptom list from the VAERS dataset, which included a total of 4331 unique symptoms. We then ranked these symptoms based on their frequency of occurrence. Similarly, we conducted keyword extraction using the symptom list in the Twitter dataset to identify and rank the most frequently mentioned symptoms. A meticulous manual review was conducted to omit symptoms extraneous to our study, such as “SARS-COV-2 TEST NEGATIVE” and “INCORRECT DOSE ADMINISTERED,” from VAERS. Consequently, two distinct tables were created to display the top 20 most frequent symptoms from both the VAERS and Twitter datasets, thereby enabling a comparative analysis.

### LDA topic modeling

The LDA Topic Modeling was utilized to discern the thematic structures in discussions about mRNA vaccines and therapeutics within our Twitter dataset; topic modeling is an unsupervised machine-learning technique that enables the discovery of latent thematic patterns in extensive text collections^47^. The LDA approach, frequently used for summarizing topics in social media data^48,49^, assumes that documents (in this case, tweets) can be represented as probabilistic distributions over a set of topics, where each topic is a probabilistic distribution of terms (words). For our analysis, we employed the Python-based Gensim library to conduct LDA topic modeling.

## Supplementary information

Supplementary Information

## Data Availability

The datasets generated and/or analyzed during the current study are available at: https://drive.google.com/drive/folders/12QR04mBYRZyPafsfSSYTBP07QMhMlYNY?usp=sharing.

## Code availability

The custom code used for sentiment and content analysis was written in Python and is deposited in GitHub: https://github.com/RyanXujiaxiang/mRNA-Result.git.
