# Supplementary material for: Mapping global public perspectives on mRNA vaccines and therapeutics
Source: NPJ Vaccines. 2024 Nov 14;9:218. doi: 10.1038/s41541-024-01019-3 (PMC11564657; doi:10.1038/s41541-024-01019-3)
Supplement: Supplementary file 1 — Supplementary Information [file 41541_2024_1019_MOESM1_ESM.pdf]

Mapping Global Public Perspectives on mRNA Vaccines and Therapeutics

Supplementary Materials

Supplementary Table 1. Performance Metrics for GPT Model Content Classification: Precision, Recall, and F1-Score.

Category	Model	Precision	Recall	F1-score
Sentiment	GPT-3.5-Turbo	0.907	0.904	0.905
Safety	GPT-3.5-Turbo	0.752	0.735	0.736
Effectiveness	GPT-3.5-Turbo	0.788	0.697	0.716
Importance	GPT-3.5-Turbo	0.840	0.789	0.800
Trust	GPT-3.5-Turbo	0.768	0.735	0.740

Supplementary Note 1. Manual Coding/GPT classification definition and prompt.

Prompt =

"""

I'm monitoring social media discussions to understand public attitudes towards the mRNA technology / vaccine. Please help me classify the provided Twitter post about the mRNA technology / vaccine.

Here are the definition of safety, effectiveness, importance and trust in authority

(1) Safety:

- Safe: mRNA technology / vaccine is safe and reliable (with no adverse reactions, etc.)
- Unsafe: Doubt about the safety of the mRNA technology / vaccine, or believe that the mRNA technology / vaccine is unsafe (including concerns about possible adverse reactions, or damage to health, etc.)

(2) Effectiveness:

- Effective: mRNA technology / vaccine is effective, to produce antibodies, or to prevent COVID-19 (or other disease), etc. (positive attitudes towards effectiveness)
- Ineffective: Doubt about the effectiveness of the mRNA technology / vaccine, or believe that the mRNA technology / vaccine is ineffective, unable to produce antibodies or prevent COVID-19 (or other disease), etc. (negative attitudes towards of effectiveness)

(3) Importance

- Important: mRNA technology / vaccine is important, necessary or needed
- Unimportant: mRNA technology / vaccine is unimportant, unnecessary or unneeded

(4) Trust in authority

- Trust: Trust in government or policy-makers (including all-level government, ministry of health, CDC, etc)
- Distrust: Doubt or distrust in government or policy-makers (including all-level government, ministry of health, CDC, etc)

The desired output should be in JSON format:

```
{
   "sentiment": "positive/neutral/negative",
   "safety": "safe/unsafe/irrelevant",
   "effectiveness": "effective/ineffective/irrelevant",
   "importance": "important/unimportant/irrelevant",
   "trust": "trust/distrust/irrelevant"
}
```

"""
